# Nonlinear dynamics and multiscale mechanisms of deep brain stimulation

**DOI:** 10.3389/fnins.2026.1778894

**Published:** 2026-02-06

**Authors:** Yue Yuan, Hao Yan, Kun Zhang, Zheshan Guo, Zhaoxiang Wang

**Affiliations:** 1 Key Lab of Biomedical Engineering for Ministry of Education, College of Biomedical Engineering and Instrument Science, Zhejiang University, Hangzhou, Zhejiang, China; 2 ZhejiangLab, Research Center for Life Sciences Computing, Hangzhou, Zhejiang, China; 3 State Key Laboratory of Digital Medical Engineering, Key Laboratory of Biomedical Engineering of Hainan Province, School of Biomedical Engineering, Hainan University, Sanya, China; 4 Sanya Research Institute of Hainan University, Sanya, Hainan, China

**Keywords:** deep brain stimulation, network neuromodulation, neural synchrony, nonlinear dynamics, state-space dynamics

## Abstract

Deep brain stimulation (DBS) is an established treatment for movement disorders and an expanding therapy for several neuropsychiatric conditions, yet its mechanisms of action remain incompletely understood. Early interpretations largely relied on linear and focal models, framing DBS as local excitation, inhibition, or a reversible lesion. Accumulating evidence, however, indicates that DBS reorganizes neural activity across multiple spatial and temporal scales, engaging distributed circuits and network-level dynamics. Here, we synthesize experimental, computational, and clinical findings supporting a nonlinear dynamical perspective on DBS. Within this framework, pathological brain states, such as excessive β synchrony in Parkinson’s disease or hypersynchronous epileptic activity, can be conceptualized as maladaptive network regimes. DBS perturbs these regimes in a state-dependent manner, disrupting pathological synchrony, modulating intrinsic oscillations, inducing threshold-like state transitions, and, in some contexts, altering temporal complexity. This perspective helps explain why DBS effects depend on ongoing brain state and why modest changes in stimulation timing or pattern can produce disproportionate clinical effects. Rather than prescribing specific technologies, nonlinear dynamics provides an integrative framework for interpreting diverse DBS phenomena and for understanding the principles underlying adaptive, temporally patterned, and individualized neuromodulation strategies. Together, these insights position DBS as a state-dependent, network-level intervention operating within a nonlinear brain, complementing classical mechanisms and offering a unified lens through which to interpret its diverse therapeutic effects.

## 1 Introduction

Deep brain stimulation (DBS) has emerged as a major therapeutic advance for neurological and psychiatric disorders characterized by dysfunctional neural circuits (Sandoval-Pistorius et al., 2023). Since its clinical adoption in the 1990s, DBS has been applied to modulate distributed brain circuits underlying motor, cognitive, and affective functions, most prominently in Parkinson’s disease, essential tremor, and dystonia, with expanding applications in epilepsy and selected psychiatric conditions (Lozano et al., 2019; Yu et al., 2019; Li and Cook, 2018; Zhang et al., 2024; Krauss et al., 2021). Unlike ablative procedures, DBS provides adjustable and reversible neuromodulation through chronically implanted electrodes, enabling therapeutic intervention of pathological network activity (Lozano et al., 2019; Krauss et al., 2021). Although empirically optimized stimulation parameters, typically high-frequency stimulation (HFS), can produce robust and durable clinical benefits, the mechanisms through which DBS restores circuit function remain only partially understood (Sandoval-Pistorius et al., 2023; Shea et al., 2025).

Historically, DBS was interpreted within local and largely linear control models (Vitek, 2002; Herrington et al., 2016; Miocinovic et al., 2013). In the basal ganglia “rate model,” Parkinsonian symptoms were attributed to excessive activity within specific nuclei (e.g., subthalamic nucleus, STN), and HFS was therefore viewed as a reversible lesion that suppressed pathological activity via depolarization block or recruitment of local inhibitory afferents (Miocinovic et al., 2013; Chiken and Nambu, 2016; Albin et al., 1995; Filali et al., 2004). However, experimental and clinical studies demonstrated that such focal mechanisms cannot fully explain DBS effects (Florence et al., 2016). Stimulation can alter firing patterns in downstream neurons, evoke antidromic activity in cortical pathways, and reshape oscillatory synchronization across distributed networks (Hashimoto et al., 2003; Leblois et al., 2010; Jakobs et al., 2019). Moreover, clinical improvement correlates more closely with modulation of pathological β synchrony than with simple rate suppression, motivating a shift from purely local explanations toward circuit-level models of DBS action (Bronte-Stewart et al., 2009; Mathiopoulou et al., 2024). Notably, this conceptual shift has paralleled advances in DBS technology, as recent sensing-enabled and adaptive systems introduce explicit state dependence and temporal structure that challenge purely static, linear interpretations (Table 1).

**Table 1:** Timeline of technological development in DBS.

Year/period	Milestone	Conceptual significance
1947–1970s	Foundations of stereotactic neurosurgery and chronic stimulation	Enabled precise targeting and long-term neuromodulation, establishing DBS as a controllable intervention
1987	HFS for tremor	Introduced HFS as a reversible alternative to lesions, shaping early linear and rate-based interpretations
1990s	STN and GPi identified as DBS targets in PD	Anchored DBS within basal ganglia circuit models and rate-based pathophysiology
1997–2002	FDA approval for tremor and Parkinson’s disease	Thalamic stimulation for essential tremor and Parkinsonian tremor (1997) STN/GPi stimulation for advanced PD symptoms (2002)
2013	Responsive/closed-loop stimulation	Introduced state dependence and feedback, challenging purely linear descriptions
2015	Directional leads	Enabled spatial selectivity, highlighting network-level rather than focal effects
2020–2024	Sensing-enabled and adaptive DBS systems	Facilitated real-time monitoring and adaptive control, motivating dynamical and nonlinear frameworks

Together, these findings establish DBS as a fundamentally circuit-level intervention, setting the stage for viewing stimulation not only as a modulator of firing patterns, but as a perturbation applied to an evolving network state (Neumann et al., 2023; Ma and Tang, 2017). HFS imposes a temporally structured drive that interacts with ongoing network dynamics, regularizing disordered firing and altering excitatory–inhibitory balance (Filali et al., 2004; Johnson et al., 2020; Reese et al., 2011; Yu et al., 2018). At the population level, DBS suppresses excessive rhythmic synchronization, particularly β activity in Parkinson’s disease, while promoting more flexible network states (Yu et al., 2018; Wilson and Moehlis, 2015; Rubin and Terman, 2004). These effects propagate across hierarchically organized neural circuits through both orthodromic and antidromic pathways (Neumann et al., 2023). Thus, therapeutic benefit likely reflects coordinated changes across spatial and temporal scales that reshape information flow within pathological networks, rather than a single dominant mechanism.

## 2 Classical mechanisms of deep brain stimulation

### 2.1 Local suppression and excitation–inhibition models

Early mechanistic accounts of DBS emphasized focal excitation or inhibition within the stimulated nucleus (Vitek, 2002). In the classical basal ganglia rate model, these hypotheses proposed that therapeutic benefit reflected functional inactivation of an overactive structure (Filali et al., 2004; Dostrovsky et al., 2000). In Parkinson’s disease, hyperactivity of STN was thought to increase inhibitory output from the globus pallidus internus (GPi), thereby suppressing thalamocortical drive. HFS of the STN or GPi was therefore viewed as a reversible lesion, silencing local neurons through depolarization block or synaptic failure (Chiken and Nambu, 2016; Filali et al., 2004; Schor et al., 2022). This interpretation was supported by pharmacological inactivation studies and by observations that subsets of neurons near the electrode reduce their firing during stimulation (Filali et al., 2004; Florence et al., 2016; Dostrovsky et al., 2000; Levy et al., 2001).

However, purely local inhibition cannot account for several key observations. Effective STN stimulation can increase GPi firing, contrary to predictions of simple STN suppression (Reese et al., 2011; Jiruska et al., 2010). DBS can also simultaneously silence somatic activity while activating afferent and efferent axons (Jiruska et al., 2010; Deniau et al., 2010; Jantz and Watanabe, 2013), indicating that stimulation reorganizes rather than eliminates neural activity. These findings revealed that lesion-like mechanisms capture only a subset of DBS effects and motivated broader circuit-level interpretations (Deniau et al., 2010).

### 2.2 Output regularization and disruption of pathological transmission

To resolve these inconsistencies, attention shifted from local suppression to models emphasizing disruption of pathological signaling (Chiken and Nambu, 2016; Grill et al., 2004). The informational lesion hypothesis proposed that DBS overrides abnormal activity patterns rather than simply reducing firing (Schor et al., 2022; Meissner et al., 2005). In this framework, HFS regularizes axonal output: while somatic firing near the electrode may decrease, stimulated axons are driven to fire in a highly regular, time-locked pattern (Reese et al., 2011; Agnesi et al., 2015). This imposed pattern masks endogenous pathological signals and prevents their propagation through the network (Chiken and Nambu, 2016; Reese et al., 2011; Wang et al., 2018; Feng et al., 2017; Chiken and Nambu, 2014).

Consistent with this view, clinical improvement correlates weakly with changes in mean fire rate but more strongly with disruption of abnormal temporal structure, particularly pathological oscillations (Chiken and Nambu, 2016; Chiken and Nambu, 2014). By imposing a regular high-frequency drive, often described as “jamming,” DBS functionally decouples the target nucleus from the broader circuit, creating an informational lesion even when neurons remain active (Florence et al., 2016; Hashimoto et al., 2003; Leblois et al., 2010; Meissner et al., 2005). This emphasis on temporal structure marked a conceptual advance beyond purely excitatory–inhibitory accounts.

### 2.3 Network propagation and antidromic recruitment

If DBS regularizes axonal output, its effects are expected to propagate beyond the stimulation site. Indeed, DBS produces network-wide consequences through both orthodromic and antidromic activation of axonal pathways (Malekmohammadi et al., 2018; McConnell et al., 2012; Kang and Lowery, 2014). STN stimulation influences downstream basal ganglia structures while simultaneously driving antidromic activity in upstream cortical projections (Johnson et al., 2020; Li et al., 2012). Short-latency cortical responses observed in animal and human studies provide direct evidence for rapid recruitment of cortico-subthalamic projections (Johnson et al., 2020; Li et al., 2012; Miocinovic et al., 2018).

In addition, DBS can also recruit axonal collaterals, producing divergent effects across multiple downstream targets (Hammond et al., 2008; Anderson et al., 2018). For example, STN stimulation modulates pallidal output while simultaneously engaging brainstem and cortical modulatory systems, leading to widespread transmitter release and circuit reconfiguration (Anderson et al., 2018; Bar-Gad et al., 2004). These findings establish DBS as a network-level intervention whose effects depend on the embedding circuitry, rather than a purely focal manipulation of a single nucleus (Sobesky et al., 2022; McIntyre and Hahn, 2010).

### 2.4 Limitations of classical linear models

Together, classical models identify several experimentally supported mechanisms: local inhibitory effects, axonal activation and output regularization, disruption of pathological transmission, and distributed network engagement (summarized in Table 2) (Filali et al., 2004; Reese et al., 2011; Dostrovsky et al., 2000; Chiken and Nambu, 2014; Malekmohammadi et al., 2018; McConnell et al., 2012; Dorval et al., 2010; Rosenbaum et al., 2014; Lee et al., 2011). Each of these mechanisms is experimentally supported and likely contributes under specific conditions. However, DBS effects vary substantially across disorders, brain states, and stimulation parameters, and cannot be fully explained by any single mechanism in isolation. Instead, therapeutic outcomes appear to depend on how these mechanisms interact and are expressed within the broader network (McIntyre and Anderson, 2016; Wu et al., 2021; Gittis and Sillitoe, 2024).

**Table 2:** Proposed deep brain stimulation mechanisms.

Descriptive level	Core interpretation	Representative evidence
Local neuronal effects	HFS suppresses or alters activity near the electrode, mimicking a reversible lesion	Reduced firing in subsets of neurons; symptom relief after focal inactivation
Axonal output shaping	DBS regularizes axonal output despite variable somatic firing	Stimulus-locked axonal firing; weak correlation between mean firing rate and clinical benefits
Network propagation	DBS-evoked effects propagate through connected circuits via orthodromic and antidromic pathways	Cortical responses to STN-DBS; modulation of distributed basal ganglia–cortical networks
Dynamical state modulation	DBS perturbs global network dynamics in a state-dependent manner	Changes in synchrony, oscillatory structure, and variability across brain states

From this perspective, the limitations of classical accounts arise not from the absence of relevant mechanisms, but from the difficulty of describing how their interactions evolve across different system states (Lozano et al., 2019; McIntyre and Hahn, 2010; Neumann et al., 2023). Classical models primarily characterize local or circuit-level processes, yet offer limited insight into why DBS efficacy often exhibits strong state dependence, threshold-like transitions, and sensitivity to stimulation history (Wu et al., 2021; Neumann et al., 2023; Dovzhenok et al., 2012; Zheng et al., 2020). Importantly, these mechanisms are therefore not rendered obsolete by higher-level dynamical descriptions; rather, they constitute the substrates through which stimulation acts, while a nonlinear dynamical perspective captures how their combined effects are integrated across time and network state (Lozano et al., 2019; Wilson and Moehlis, 2015; McIntyre and Hahn, 2010; Gittis and Sillitoe, 2024; Neumann et al., 2023). This view motivates treating DBS as a perturbation applied to a complex dynamical system, capable of reshaping pathological brain states toward more adaptive regimes.

## 3 Nonlinear mechanisms of neuromodulation: synchrony, rhythms, and complex dynamics

Whereas classical models emphasize local effects on individual neurons or nuclei, a nonlinear-dynamics perspective considers how DBS alters the global state of distributed networks (McIntyre and Hahn, 2010; Breakspear, 2017). HFS often evokes system-level responses that cannot be reduced to a linear sum of single-cell effects. Instead, DBS disrupts pathological synchrony, reorganizes intrinsic oscillations, and reshapes coordinated activity across spatial and temporal scales (Chiken and Nambu, 2016; Schor et al., 2022; De Hemptinne et al., 2015; Wilson et al., 2011). In this framework, DBS acts in a state-dependent manner, with outcomes shaped by ongoing network activity and stimulation timing (Cagnan et al., 2017; Little et al., 2013).

In the following sections, we outline four interrelated nonlinear processes through which these effects can be described: (i) disruption of pathological synchrony (McConnell et al., 2012; Brown, 2007); (ii) modulation of intrinsic oscillations (Rubin and Terman, 2004; De Hemptinne et al., 2015; Ma et al., 2019); (iii) induction of network state transitions via bifurcation-like mechanisms (Breakspear, 2017; Wang et al., 2022; Gu et al., 2015); and (iv) restoration of long-range temporal correlations (LRTC) and dynamical complexity (Hohlefeld et al., 2012; Wang et al., 2024). These descriptions are not competing mechanisms but complementary analytical perspectives operating at different levels, from population synchrony and rhythmic structure to state-space organization and multiscale temporal integration. Overlaps between them therefore reflect differences in descriptive level rather than distinct causal explanations.

Although many illustrative examples are drawn from Parkinson’s disease, the nonlinear principles discussed here are not disease-specific. Instead, disease specificity arises from differences in pathological network organization, stimulation targets, and paradigms (Jakobs et al., 2019; Koeglsperger et al., 2019). While distinct conditions may benefit from alternative stimulation strategies, such as irregular or stochastic inputs, the present review focuses on principles associated with HFS, the most widely applied clinical paradigm (Lozano et al., 2019; Wagle Shukla et al., 2017).

### 3.1 Disrupting pathological synchrony

At the level of population coherence, excessive neuronal synchrony is a core pathophysiological feature of several brain disorders, including Parkinson’s disease and epilepsy (Uhlhaas and Singer, 2006). In Parkinson’s disease, bradykinesia and rigidity are closely linked to exaggerated β-band synchrony within basal ganglia–cortical circuits (Brown, 2007; Hammond et al., 2007), whereas epileptic seizures arise from abrupt, hypersynchronous discharges across large neuronal populations (Jiruska et al., 2013). From a nonlinear-dynamics perspective, these excessively coherent patterns can be viewed as pathological attractors: self-stabilizing network states that are resistant to perturbation (Breakspear, 2017; Deco and Jirsa, 2012). A principal goal of DBS is therefore to destabilize these synchronous attractors and promote more flexible, desynchronized network dynamics (McIntyre and Hahn, 2010; De Hemptinne et al., 2015; Wilson et al., 2011).

Converging evidence indicates that DBS achieves therapeutic benefit primarily by disrupting pathological synchrony rather than simply suppressing neural activity (Kromer and Tass, 2020; Qasim et al., 2016; Medeiros and Moraes, 2014; Kuhn et al., 2004). In Parkinson’s disease, effective STN stimulation acutely reduces β-band power within the STN and connected cortical regions, and the magnitude of β suppression closely parallels clinical improvement (Kromer and Tass, 2020; Qasim et al., 2016; Medeiros and Moraes, 2014; Kuhn et al., 2004). Similar reductions following dopamine-replacement therapy reinforce the view that excessive β synchrony reflects a core network abnormality (Mathiopoulou et al., 2024). Previous studies further suggest that HFS shifts STN activity from rhythmic synchrony to irregular, asynchronous firing, largely through asynchronous GABAergic input from the external globus pallidus (Wilson and Moehlis, 2015; Koeglsperger et al., 2019; Xu et al., 2025). Thus, symptom relief appears to arise from normalization of aberrant β synchrony, rather than compensation for dopamine loss alone (Mathiopoulou et al., 2024; Wilson and Moehlis, 2015; Xu et al., 2025; Eusebio et al., 2011; Moran et al., 2012; Tass and Hauptmann, 2007).

Comparable desynchronizing effects are observed in epilepsy. In hippocampal models, typically studied in anesthetized rats, HFS suppresses hypersynchronous epileptiform discharges even when overall activity levels remain similar: neurons continue to fire, but in a disorganized, non-bursting manner that lacks seizure-like coherence (Wang et al., 2021). Although hippocampal circuitry differs from basal ganglia networks, these findings illustrate a general stimulation principle whereby axonal activation disrupts pathological signal propagation and decouples downstream neuronal populations. Consistent with this view, clinical recordings show that anterior thalamic DBS produces frequency-dependent desynchronization, with stimulation >45 Hz reducing hippocampal and cortical synchrony and suppressing epileptiform events (Yu et al., 2018). Moreover, spatially distributed or temporally irregular stimulation can enhance seizure suppression, consistent with a synchrony-disruption mechanism (de Oliveira et al., 2018; Arcot Desai et al., 2014).

Together, these findings support a unifying framework in which DBS acts by destabilizing maladaptive synchronous states, such as β oscillations in Parkinson’s disease or hypersynchronous discharges in epilepsy, and shifting networks toward more irregular yet functionally stable regimes (Wilson and Moehlis, 2015; Wilson et al., 2011). Importantly, DBS does not simply silence neural activity; rather, it selectively disrupts pathological coherence while preserving healthier asynchronous dynamics, distinguishing it from classical inhibitory models.

### 3.2 Oscillatory modulation and rhythmic network intervention

At the level of rhythmic organization, brain function is fundamentally organized by oscillations across multiple frequency bands, and many neurological disorders are marked by abnormalities in these rhythms (Mathiopoulou et al., 2024; Buzsáki, 2006). In Parkinson’s disease, for example, β-band activity becomes excessively prominent within motor circuits and is widely regarded as a network signature of bradykinesia and rigidity (Malekmohammadi et al., 2018; Brown, 2007). DBS directly perturbs these rhythms and oscillatory dynamics. Clinical recordings show that STN-DBS rapidly suppresses β power in both the STN and motor cortex, with the degree of suppression closely paralleling clinical improvement (Bronte-Stewart et al., 2009; Mathiopoulou et al., 2024). Importantly, the oscillatory effects of DBS differ partly from those of dopaminergic therapy: while levodopa preferentially reduces low-β synchrony, DBS tends to suppress β activity more broadly and can, in some patients, induce stimulation-locked high-frequency oscillations consistent with circuit entrainment (Hashimoto et al., 2003; Mathiopoulou et al., 2024; McConnell et al., 2012; Cheyne, 2013; Priori et al., 2004; Johnson et al., 2008).

DBS also modulates rhythms beyond the β range. In essential tremor, thalamic stimulation suppresses tremor-related oscillations at ~4–6 Hz (Benabid et al., 1991; Milosevic et al., 2018). In epilepsy, DBS can disrupt hypersynchronous epileptiform activity and promote faster, lower-amplitude rhythms associated with more stable network states (Fisher et al., 2010; Fisher, 2023). Experimental studies further demonstrate that HFS can markedly suppress low-frequency oscillations, including hippocampal theta activity (4–8 Hz) during CA1 stimulation and ~9-Hz rhythms in the globus pallidus externus (GPe) and substantia nigra pars reticulata (SNr) during STN stimulation, with corresponding reductions in theta-locked spiking and local-field-potential power (Agnesi et al., 2015; McConnell et al., 2012; Ma et al., 2019). These effects reflect restructuring of temporal organization, not merely rate changes: spike timing becomes decoupled from dominant rhythms, indicating coordinated modulation across cellular and population levels. Consistent with this view, coordinated-reset stimulation deliberately applies spatiotemporally patterned inputs to disrupt pathological synchrony and produce longer-lasting desynchronization (Popovych and Tass, 2012; Fan and Wang, 2015).

Taken together, these findings support a frequency-domain account of DBS: stimulation acts as a network-level intervention on oscillatory dynamics, suppressing pathological synchrony, restoring physiological rhythmic organization, and recalibrating cross-frequency interactions (McConnell et al., 2012; McIntyre and Hahn, 2010; Scherer et al., 2020; Xiao et al., 2018). Crucially, therapeutic benefit depends not only on stimulation intensity but on how stimulation interacts with ongoing intrinsic rhythms—a hallmark of nonlinear dynamical control.

### 3.3 Bifurcation-like state transitions and critical dynamics

At the level of state-space dynamics, DBS can be viewed as a driver of transitions between distinct network states (Breakspear, 2017). When neural circuits operate near critical points, small perturbations can precipitate abrupt and distinct changes in network dynamics, analogous to mathematical bifurcations (De Maesschalck and Wechselberger, 2015). Many pathological brain states can therefore be conceptualized as attractors in network state space, such as the stable β-oscillatory regime in Parkinson’s disease or recurrent epileptiform discharges in epilepsy (Hammond et al., 2007; Saggio et al., 2020; Jirsa et al., 2014; Brittain and Brown, 2014). HFS can be regarded as an external forcing input that displaces the system from these attractors and, under suitable conditions, carries it across a critical threshold into a more physiological regime (Rubin and Terman, 2004; McIntyre and Hahn, 2010).

Computational work strongly supports this interpretation. The chaotic desynchronization hypothesis proposes that appropriately patterned HFS destabilizes a pathologically synchronized population, pushing it into a high-dimensional, irregular regime that abolishes the coherent rhythm sustaining symptoms (Wilson and Moehlis, 2015; Rubin and Terman, 2004; Wilson et al., 2011). Related principles underlie coordinated-reset stimulation, in which pulses delivered at distinct phases fragment a synchronized population into weakly coupled clusters, inducing longer-lasting desynchronization via plasticity-dependent reorganization (Fan and Wang, 2015; Wang and Wang, 2017; Wang et al., 2016; Tass, 2003). Experimental evidence further reveals threshold-like transitions and abrupt changes in firing patterns under sustained HFS, consistent with bifurcation-like dynamics observed *in vivo* and attributable to nonlinear axonal and network mechanisms (Wang et al., 2022; Yuan et al., 2025). In hippocampal epilepsy models, brief HFS can precipitate after-discharges, whereas longer trains delivered at the same intensity suppress seizures entirely, indicating a nonlinear dependence on stimulation duration, whereby prolonged stimulation carries the system beyond the seizure bifurcation into a more stable, non-seizing state (Wang et al., 2021; Saggio et al., 2020; Lesser et al., 1999).

More recent electrophysiological studies indicate that DBS interacts with intrinsic nonlinearities rather than acting as a purely linear drive (McIntyre and Hahn, 2010; McIntyre et al., 2004). Within clinically relevant frequency ranges, subtle changes in inter-pulse interval (IPI) or amplitude can push the system across critical thresholds, producing abrupt transitions between qualitatively distinct activity patterns, including alternating regimes of sustained firing and quiescence (Wang et al., 2022; Yuan et al., 2025; Zhang et al., 2020; Hu et al., 2023; Zheng et al., 2021). Strikingly, even when mean frequency and pulse count are held constant, re-ordering the intervals can markedly alter responses, indicating proximity to bifurcation points where fine temporal structure determines the emergent state (Zheng et al., 2020; Grill, 2018; Hess et al., 2013). Modeling studies attribute this sensitivity to nonlinear recovery of voltage-gated sodium channels and activity-dependent potassium accumulation, giving rise to intermittent conduction failure and bistability (Zheng et al., 2020; Yuan et al., 2025; Yang et al., 2006; Gu and Chen, 2014). Thus, neural responses to DBS depend not simply on mean frequency but on the precise temporal pattern of stimulation, reflecting history-dependent integration in a system poised near bifurcation (Hess et al., 2013; Brocker et al., 2017).

Together, these observations suggest that DBS influences neural circuits not merely by altering mean firing rates, but by reorganizing patterns of activity across populations, leading to changes in synchrony, variability, and state stability (Krauss et al., 2021; Wang et al., 2018; Feng et al., 2017). Such effects are consistent with a reshaping of the underlying dynamical landscape of neural circuits. By pushing networks across critical thresholds and out of pathological attractor states, DBS can trigger abrupt, nonlinear state transitions that destabilize maladaptive synchrony and reset brain dynamics toward more physiological regimes.

### 3.4 Restoring complex dynamics and multiscale integration

At the level of multiscale temporal organization, healthy brain activity is scale-free and fractal, reflected in LRTC across multiple time scales (Linkenkaer-Hansen et al., 2001; Buzsáki and Mizuseki, 2014). Such correlations are often interpreted as signatures of near-critical dynamics, a regime associated with flexible information processing and multiscale coordination (Chialvo, 2010; Beggs and Timme, 2012). In EEG and MEG recordings, robust LRTC are observed in healthy individuals, whereas several neurological disorders, including Parkinson’s disease, epilepsy and disorders of consciousness, show attenuated temporal correlations, suggesting a shift toward less adaptive dynamical regimes (Hohlefeld et al., 2012; Linkenkaer-Hansen et al., 2001; Linkenkaer-Hansen et al., 2004; Bhattacharya et al., 2005; Hohlefeld et al., 2013; Liang et al., 2018).

From a nonlinear-dynamics perspective, DBS may influence these properties indirectly by disrupting excessive synchrony and preventing network activity from collapsing into low-dimensional attractors (Herrington et al., 2016; McIntyre and Hahn, 2010). Experimental studies in hippocampal and cortical preparations demonstrate that periodic or patterned stimulation can enhance downstream temporal complexity and increase the Hurst exponent, even when the stimulus itself lacks scale-free structure (Wang et al., 2024; Hohlefeld et al., 2013; Yuan et al., 2024). Clinically, changes in spectral scaling and LRTC have been reported in cortical and subcortical recordings during DBS in some patient cohorts, consistent with partial restoration of multiscale temporal organization (Hohlefeld et al., 2012; Hohlefeld et al., 2013; Lee et al., 2025).

Nevertheless, evidence for LRTC modulation remains heterogeneous and context dependent. While robust effects have been observed in cortical recordings, their stability within basal ganglia nuclei, particularly under chronic stimulation, remains less well established, and LRTC measures are sensitive to recording duration, noise, and behavioral state (Hohlefeld et al., 2012; Linkenkaer-Hansen et al., 2001; Darbin et al., 2006). Moreover, although metrics such as the Hurst exponent provide valuable insight into statistical structure and dynamical regime, they are not currently practical as real-time control variables in clinical DBS, where simpler biomarkers such as β-band power or burst dynamics are more readily measurable (Hohlefeld et al., 2012; Dimitriadis and Linden, 2016; Nikulin et al., 2012).

Accordingly, restoration of temporal complexity should be viewed not as a primary mechanism of DBS, but as a higher-level descriptor of how network dynamics reorganize following stimulation. Within this framework, LRTC and related measures offer a complementary lens for understanding how DBS reshapes information processing across time scales, rather than a replacement for established rate- or oscillation-based accounts (Hohlefeld et al., 2012; Wang et al., 2024; Hohlefeld et al., 2013). This perspective highlights both the promise and the current limitations of applying nonlinear dynamical metrics to clinical neuromodulation. Accordingly, changes in temporal complexity and LRTC should be interpreted as descriptive markers of network reorganization rather than as disease-specific or target-specific mechanisms of DBS.

## 4 Clinical implications and technological outlook

Viewing DBS through a nonlinear dynamics lens emphasizes its state-dependent interaction with ongoing network activity rather than portraying stimulation as a uniform high-frequency pacemaker (McIntyre and Hahn, 2010; Breakspear, 2017). The therapeutic goal becomes stabilizing networks within a regime that preserves flexibility while preventing pathological synchrony. Importantly, this perspective does not replace established rate- or oscillation-based accounts but provides a complementary framework for understanding why identical stimulation parameters can yield different outcomes across brain states.

From this viewpoint, closed-loop or adaptive DBS can be interpreted as a practical implementation of state-dependent intervention. In current clinical systems, adaptive stimulation is typically triggered by readily measurable biomarkers such as β-band power or β-burst dynamics (Little et al., 2013; Priori et al., 2013), without requiring explicitly nonlinear metrics. Nonlinear theory does not mandate such approaches, but helps explain why timing stimulation to emerging pathological states can be more effective than continuous delivery, by selectively perturbing networks when they approach maladaptive regimes.

A second implication concerns temporal patterning. Experimental and modeling studies demonstrate that neural circuits integrate inputs nonlinearly, such that stimulation timing and pattern can critically shape network responses even when mean frequency is held constant (Grill, 2018; Hess et al., 2013). Strategies such as stochastic IPI, phase-specific stimulation, multi-frequency inputs or coordinated-reset protocols may selectively disrupt pathological rhythms while limiting entrainment (Popovych and Tass, 2012; Tass, 2003; Grill, 2018; Hoang et al., 2017). DBS is therefore better viewed as precision temporal modulation, not a fixed paradigm (Krauss et al., 2021).

Finally, personalization of neuromodulation reflects inter-individual differences in anatomy, connectivity, and baseline dynamics, motivating patient-specific targeting and parameter selection (Neumann et al., 2023; Horn et al., 2020). Such personalization can be achieved within linear or biophysical frameworks, including digital twin models. Nonlinear perspectives contribute by offering additional insight into differences in network stability, sensitivity to perturbation, or proximity to critical transitions, and are therefore best viewed as complementary tools for interpretation and offline optimization rather than mandatory control principles.

Together, these considerations position nonlinear dynamics as an interpretive framework that enriches, rather than dictates, technological development. In practice, robust biomarkers such as β-band activity remain central to clinical implementation, while dynamical metrics may assist in mechanistic understanding and parameter optimization.

## 5 Conclusion

DBS has evolved from being viewed primarily as a balance between excitation and inhibition to a network-level intervention acting within a complex, nonlinear brain. A nonlinear-dynamical perspective complements classical mechanisms by framing DBS as a state-dependent perturbation that disrupts maladaptive synchrony, reshapes network dynamics, and preserves functional flexibility. This framework helps explain why modest changes in stimulation timing or pattern can yield disproportionate effects and why clinical outcomes depend on the state of the brain.

Rather than implying a shift in therapeutic goals or technologies, this perspective provides an integrative lens for understanding how diverse DBS effects emerge across scales. In this view, DBS is best understood not as a means of simple rate suppression, but as an intervention that stabilizes pathological networks while maintaining adaptive variability, supporting restoration of healthy network function rather than enforcing rigid control.
